# Deep Learning Classification of Drusen, Choroidal Neovascularization, and Diabetic Macular Edema in Optical Coherence Tomography (OCT) Images

**DOI:** 10.7759/cureus.41615

**Published:** 2023-07-09

**Authors:** Parsa Riazi Esfahani, Akshay J Reddy, Neel Nawathey, Muhammad S Ghauri, Mildred Min, Himanshu Wagh, Nathaniel Tak, Rakesh Patel

**Affiliations:** 1 Medicine, California University of Science and Medicine, Colton, USA; 2 Ophthalmology, California Northstate University, Rancho Cordova, USA; 3 Neurosurgery, California University of Science and Medicine, Colton, USA; 4 Dermatology, California Northstate University College of Medicine, Elk Grove, USA; 5 Medicine, California Northstate University College of Medicine, Elk Grove, USA; 6 Medicine, Arizona College of Osteopathic Medicine, Midwestern University, Glendale, USA; 7 Internal Medicine, East Tennessee State University, Quillen College of Medicine, Johnson City, USA

**Keywords:** deep learning artificial intelligence, optical coherence tomography (oct), oct, diabetic macular edema, deep learning, choroidal neovascularization, drusen

## Abstract

Background

Age-related macular degeneration (AMD), diabetic retinopathy (DR), drusen, choroidal neovascularization (CNV), and diabetic macular edema (DME) are significant causes of visual impairment globally. Optical coherence tomography (OCT) imaging has emerged as a valuable diagnostic tool for these ocular conditions. However, subjective interpretation and inter-observer variability highlight the need for standardized diagnostic approaches.

Methods

This study aimed to develop a robust deep learning model using artificial intelligence (AI) techniques for the automated detection of drusen, CNV, and DME in OCT images. A diverse dataset of 1,528 OCT images from Kaggle.com was used for model training. The performance metrics, including precision, recall, sensitivity, specificity, F1 score, and overall accuracy, were assessed to evaluate the model's effectiveness.

Results

The developed model achieved high precision (0.99), recall (0.962), sensitivity (0.985), specificity (0.987), F1 score (0.971), and overall accuracy (0.987) in classifying diseased and healthy OCT images. These results demonstrate the efficacy and efficiency of the model in distinguishing between retinal pathologies.

Conclusion

The study concludes that the developed deep learning model using AI techniques is highly effective in the automated detection of drusen, CNV, and DME in OCT images. Further validation studies and research efforts are necessary to evaluate the generalizability and integration of the model into clinical practice. Collaboration between clinicians, policymakers, and researchers is essential for advancing diagnostic tools and management strategies for AMD and DR. Integrating this technology into clinical workflows can positively impact patient care, particularly in settings with limited access to ophthalmologists. Future research should focus on collecting independent datasets, addressing potential biases, and assessing real-world effectiveness. Overall, the use of machine learning algorithms in conjunction with OCT imaging holds great potential for improving the detection and management of drusen, CNV, and DME, leading to enhanced patient outcomes and vision preservation.

## Introduction

Drusen, choroidal neovascularization (CNV), and diabetic macular edema (DME) are ocular conditions that present significant challenges to global eye health [1]. Age-related macular degeneration (AMD) is the leading cause of irreversible vision loss in the elderly, and small yellow deposits beneath the retina, known as drusen, are hallmark features of this condition [2, 3]. CNV is the abnormal growth of blood vessels beneath the retina and is often associated with neovascular AMD. The estimated annual incidence of AMD in the United States is around 1.47% [4]. DME is a common complication of diabetic retinopathy that causes vision impairment due to an accumulation of fluid in the macula. This disease has an incidence of 4.4% among adults in the United States with diabetes mellitus [5]. Treatments for diabetic retinopathy include laser photocoagulation, intravitreal injections of anti-vascular endothelial growth factor (VEGF) medications, and corticosteroid injections to reduce retinal inflammation and edema [6, 7]. For age-related macular degeneration (AMD), treatment options include intravitreal injections of anti-VEGF medications, which help reduce abnormal blood vessel growth and leakage, as well as photodynamic therapy (PDT) and laser photocoagulation in certain cases to target and seal leaking blood vessels. These treatments aim to slow the progression of the diseases, preserve vision, and prevent further vision loss [8, 9].

Optical coherence tomography (OCT) imaging has become a valuable diagnostic tool for evaluating drusen, CNV, and DME by providing high-resolution cross-sectional images of retinal structures [10, 11]. OCT images allow for detailed visualization of drusen morphology, identification of CNV-related changes, and quantification of macular thickness and fluid accumulation in DME [12, 13]. However, interpreting OCT images can be subjective and prone to inter-observer variability. This highlights the need for standardized diagnostic approaches. Machine learning and deep learning algorithms offer promising avenues for enhancing diagnoses by analyzing OCT images using artificial intelligence (AI) techniques. These algorithms learn intricate patterns associated with ocular conditions such as drusen morphology or CNV-related changes. By doing so, they enable more accurate detection [14].

Therefore, this study aims to develop a robust detection model for drusen, CNV, and DME, which are hallmarks of AMD and diabetic retinopathy, using OCT images and artificial intelligence (AI) techniques. We aim to revolutionize diagnosis by providing healthcare professionals with an objective, standardized tool that utilizes AI technology. The field of ophthalmology holds a great deal of promise when it comes to utilizing machine learning models. Notably, such models provide us with the ability to detect diseases earlier on while also ensuring treatments can be initiated more promptly alongside improved management strategies for treating patients effectively. As it presently stands, there are regions where highly specialized ophthalmologists remain scarce or inaccessible; therefore, integrating AI-based detection models into workflows could improve matters significantly. This study investigates how machine learning algorithms enhance diagnostic accuracy related explicitly to products such as drusen, CNV, or DME using OCT images. By leveraging advanced technologies such as AI development, we endeavored to create a dependable and robust detection model. Ultimately, our mission is to improve diagnostic efficiency in these critical eye conditions, contributing to global efforts in our fight against them.

## Materials and methods

This study aimed to develop an ocular pathology detection model using a dataset of 3,133 OCT images obtained from Kaggle.com, a popular online platform for datasets. The OCT images consisted of 710 images for CNV, 895 for DME, 725 for drusen, and 804 normal OCT images. The objective was to train the model to recognize specific patterns and features within the OCT images that indicate the presence of drusen, enabling accurate and automated detection.

The dataset was carefully curated to ensure a diverse representation of drusen characteristics, including different sizes, shapes, and distributions. The Kaggle.com platform provided a wide range of OCT images captured from various imaging devices and clinical settings, enhancing the generalizability of the developed model. The dataset was then divided into 2,507 training, 314 validation, and 312 testing images.

Google's collaboration platform was utilized for model development, providing a collaborative and efficient environment for data preprocessing, feature extraction, and model training. Machine learning techniques, such as convolutional neural networks (CNNs), were employed to leverage the power of deep learning algorithms in learning complex patterns and features associated with drusen in OCT images.

**Figure 1:**
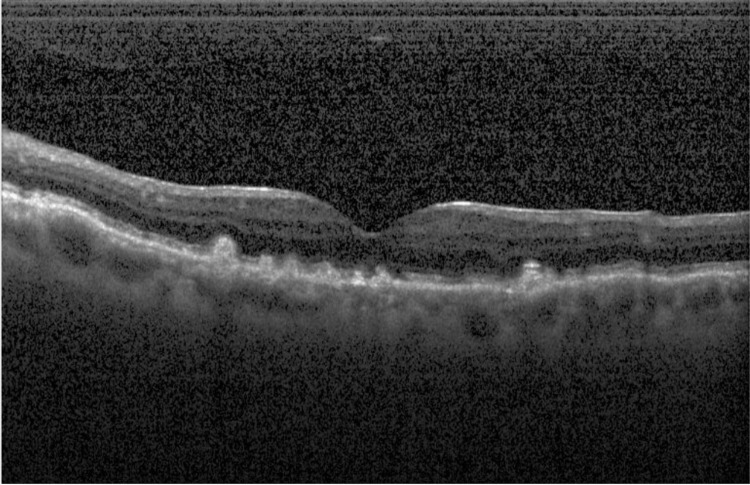
Drusen present Optical coherence tomography (OCT) image displaying drusen in the eye.

**Figure 2:**
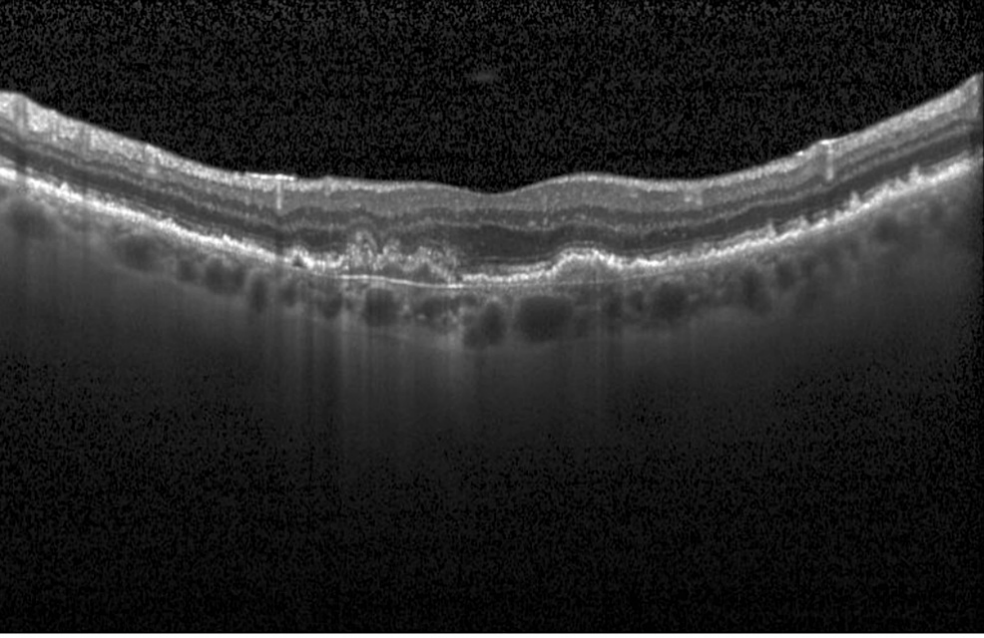
Choroidal neovascularization (CNV) present Optical coherence tomography (OCT) image from the dataset displaying CNV.

**Figure 3:**
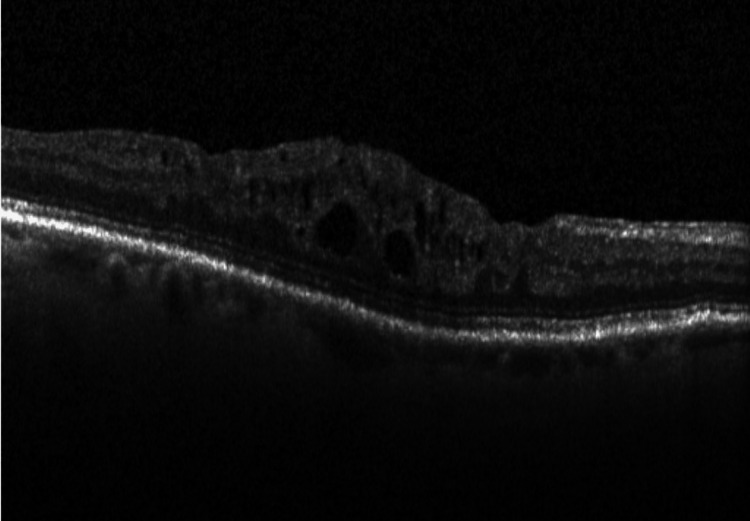
Diabetic macular edema (DME) present Optical coherence tomography (OCT) from the dataset displaying DME in the eye.

**Figure 4:**
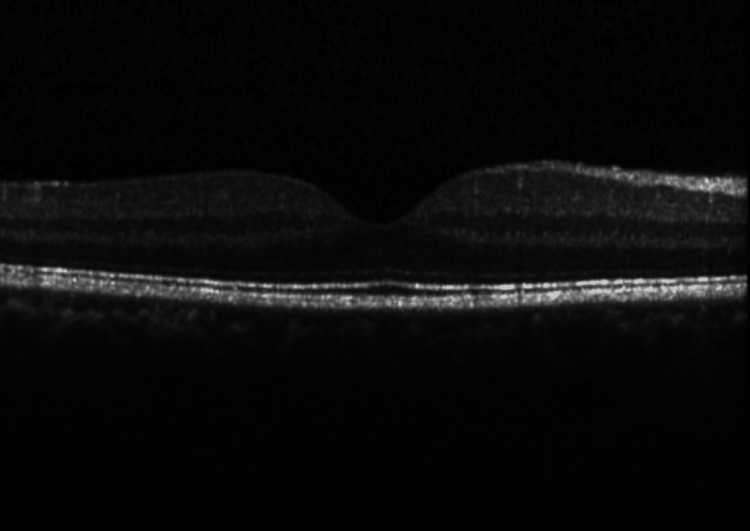
Normal retina Optical coherence tomography (OCT) image from the dataset showing a normal retina.

The model was trained using a supervised learning approach, where experienced ophthalmologists labeled the OCT images as either drusen present, CNV present, DME present, or healthy eyes (Figures 1-4).

The training process involved optimizing the model's parameters and hyperparameters to maximize its performance in disease detection.

A separate test set comprising OCT images was used to evaluate the developed model's performance. The model's accuracy, precision, recall, sensitivity, specificity, and F1 score were calculated based on its predictions on the test set. These metrics provided insights into the model's ability to accurately identify drusen-positive cases while minimizing false positives and false negatives.

## Results

In this study, we evaluated the performance of our model in detecting drusen, choroidal neovascularization (CNV), and diabetic macular edema (DME) using OCT images. The efficacy of the model is displayed using a confusion matrix, as seen in Table 1.

**Table 1:** Machine learning outcomes using the confusion matrix CNV: choroidal neovascularization; DME: diabetic macular edema

True label/Predicted label	CNV	DME	Drusen	Normal
CNV	68	3	0	0
DME	0	88	0	1
Drusen	1	3	67	1
Normal	0	1	0	79

**Figure 5:**
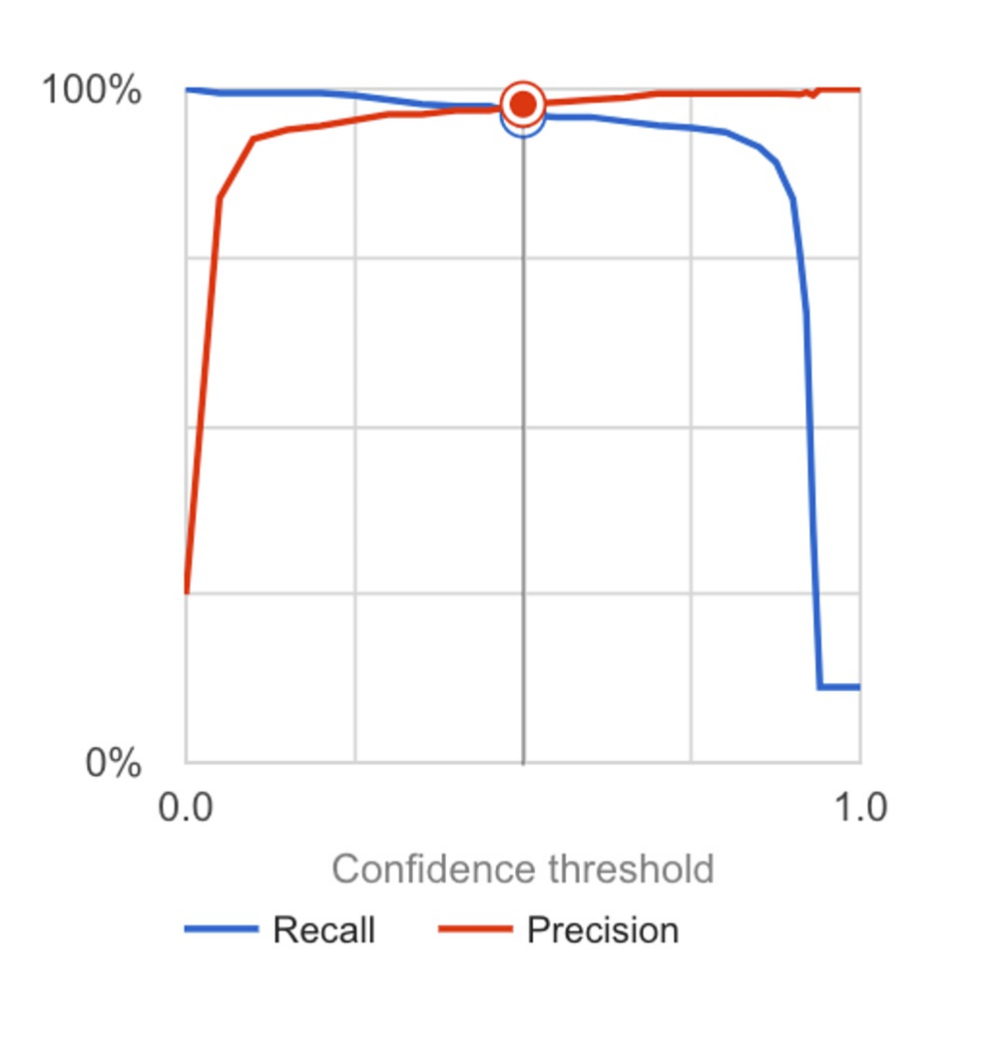
The area under the curve (AUC) graph showing the recall and precision of the model This figure displays the precision and recall of the neural network model at various confidence intervals. The authors utilized a 0.05 confidence interval to obtain data about the model.

Various confidence intervals were tested for the model, as seen in Figure 5.

However, the authors collected data from the model at a confidence interval of 0.05. The model achieved an average precision of 0.99, indicating its high precision in classifying these retinal pathologies. The precision value of 0.957 demonstrates the model's ability to accurately identify true positive cases. In contrast, the recall value of 0.962 reflects its proficiency in capturing a significant proportion of actual positive cases.

Furthermore, the model's sensitivity was calculated to be 0.985, indicating its capability to identify many true positive cases while minimizing false negatives. The specificity value of 0.987 reflects the model's ability to accurately classify true negative cases, reducing false positives. These results highlight the model's efficacy in correctly identifying both diseased and healthy OCT images.

The F1 score, which combines precision and recall, was calculated to be 0.971. This metric indicates the balance between accurate positive predictions and avoiding false positives and false negatives. The high F1 score further supports the model's performance in accurately classifying drusen, CNV, and DME cases.

The model's overall accuracy was determined to be 0.987, indicating its high accuracy in correctly classifying both positive and negative cases. This high accuracy suggests the potential of the model to aid healthcare professionals in the early detection and management of these retinal pathologies. This value, along with others, is recorded in Table 2.

**Table 2:** Model outcomes: metrics

Metrics	Values
Accuracy	0.987179487179487
Precision	0.957746478873239
Recall (sensitivity)	0.985507246376812
Specificity	0.987654320987654
F-1 score	0.971428571428571

These results underscore the potential of machine learning algorithms and OCT imaging in detecting drusen, CNV, and DME. The high precision, recall, sensitivity, specificity, F1 score, and accuracy values obtained from our model demonstrate its robustness and efficiency in distinguishing between diseased and healthy OCT images. Further validation studies and research efforts are necessary to assess the generalizability and integration of the model into clinical practice, ultimately facilitating early detection and improved management of drusen, CNV, and DME.

## Discussion

Two leading causes of visual impairment, age-related macular degeneration (AMD) and diabetic retinopathy (DR), incline toward partial or complete blindness by affecting the retina via drusen, choroidal neovascularization (CNV), and diabetic macular edema (DME) [15, 16]. Detection and management of these retinal pathologies become critical for improved patient outcomes through timely diagnosis using optical coherence tomography (OCT) imaging aided by machine learning algorithms [17].

Our study incorporated Kaggle’s OCT imaging dataset, which has 1,528 OCT training images, to develop a robust model capable of identifying CNV, DME, and drusen patterns through machine learning algorithms that perform optimally, as the Google collaboration platform recommends. Our advancement entails significant clinical implications concerning accurate classifications to provide efficient treatment options at earlier stages. Our results yield high precision and recall values for classifying diseased versus healthy OCT images while staying true to positive and negative rates via sensitivity and specificity values. This model holds the potential for tremendous value in pathology detection and monitoring. However, we acknowledge some limitations in our study. Using Kaggle's OCT Imaging dataset may not entirely represent the diversity or complexity found within retinal pathologies useful in real-world clinical settings, which could introduce unintended biases into any derived models. The initial recall for our model based on the information presented in Figure 5 was fairly high, which could potentially indicate that the model was overfitting. If the model that was trained was indeed overfitting, it could be a potential limitation to its functionality in a hospital setting. The best way to ensure this doesn't happen is to conduct further studies utilizing this model on large OCT image datasets. The model could also be improved if necessary in future studies by increasing the number of OCT images in the training set and by adjusting the learning rate of the model. In addition to this issue, there are potential influences from both the size and diversity of training data sets, along with algorithm selection and hyperparameters affecting model performance. Generalizability into full-scale clinical scenarios requires further examination.

To overcome these constraints present in the research conducted thus far while ensuring adequate robustness and reliability, validating such models across different settings and populations remains required. Plans should focus on collecting more independent datasets incorporating information sourced from several sources, including several primary institutions working together to continue pushing predictive ability boundaries forward.

Furthermore, assessing the real-world effectiveness of the developed model blends with future considerations. Our research highlights the potentiality of OCT imaging and machine learning algorithms in managing CNV, DME, and drusen detection. Additionally, it demonstrates high accuracy standards, suggesting utility in efficiently distinguishing between healthy and diseased patients concerning OCT scans.

We call for more exploration and validation efforts to ensure these promising models effectively operate beyond white-identity-focused contexts. Integrating this novel technology within clinical workflows will provide essential insights into real-world scenarios that impact patient care positively.

It remains encouraging to establish collaborative relationships between clinicians, policymakers, and researchers seeking new diagnostic tools and management-driven strategies for diabetic retinopathy and AMD.

## Conclusions

This study demonstrates the potential of machine learning algorithms for detecting drusen, CNV, and DME using OCT images. The developed model, trained on a diverse dataset of OCT images from Kaggle.com, achieved high accuracy and performance in identifying the presence of different eye pathologies. Integrating this model into clinical practice could provide ophthalmologists with an objective and efficient tool for ocular disease detection, leading to early diagnosis and tailored treatment strategies for patients with age-related macular degeneration (AMD). This technology could potentially prove helpful in a rural setting with no or minimal access to an ophthalmologist, where screenings could be done through machine learning algorithms. Further research and validation studies are warranted to assess the generalizability and scalability of the model, as well as its potential for integration into existing imaging systems. The advancement of automated drusen detection using OCT imaging holds great promise for enhancing the diagnostic capabilities and management of AMD, ultimately improving patient outcomes and preserving vision.
